# Transjugular transcatheter edge-to-edge mitral valve repair in a patient with functional mitral regurgitation: a case report

**DOI:** 10.1093/ehjcr/ytae668

**Published:** 2024-12-18

**Authors:** Yusuke Enta, Yoshiko Munehisa, Natsuko Satomi, Yukihiro Hayatsu, Norio Tada

**Affiliations:** Department of Cardiovascular Medicine, Sendai Kousei Hospital, 1-20 Tsutsumidori-amamiya, Aoba Ward, Sendai, Miyagi 9810914, Japan; Department of Laboratory Medicine, The Jikei University School of Medicine, 3-19-18 Nishi-Shimbashi, Minato Ward, Tokyo 1058471, Japan; Department of Cardiovascular Medicine, Sendai Kousei Hospital, 1-20 Tsutsumidori-amamiya, Aoba Ward, Sendai, Miyagi 9810914, Japan; Department of Cardiovascular Medicine, Sendai Kousei Hospital, 1-20 Tsutsumidori-amamiya, Aoba Ward, Sendai, Miyagi 9810914, Japan; Department of Cardiovascular Surgery, Sendai Kousei Hospital, 1-20 Tsutsumidori-amamiya, Aoba Ward, Sendai, Miyagi 9810914, Japan; Department of Cardiovascular Medicine, Sendai Kousei Hospital, 1-20 Tsutsumidori-amamiya, Aoba Ward, Sendai, Miyagi 9810914, Japan

**Keywords:** Transjugular, Right internal jugular vein, Transcatheter edge-to-edge mitral valve repair

## Abstract

**Background:**

Transcatheter edge-to-edge mitral valve repair (M-TEER) using the MitraClip system is primarily performed using the transfemoral approach. However, when this approach is not feasible, the transjugular approach can be used as an alternative.

**Case summary:**

A 57-year-old man presented with heart failure and persistent New York Heart Association class IV symptoms, refractory to guideline-directed medical therapy, intravenous therapy, and intra-aortic balloon pumping. His medical history included pulmonary embolism secondary to deep vein thrombosis, which occluded the inferior vena cava (IVC). Transthoracic echocardiography (TTE) revealed severe functional mitral regurgitation (FMR). The IVC occlusion made the transfemoral approach impossible; hence, transjugular M-TEER was planned. Transseptal puncture was performed via the right internal jugular (RIJ), 32 mm above the mitral annulus. A Confida wire was positioned in the left ventricle, and a steerable guiding catheter was introduced with 180° clockwise rotation of the +knob for septal crossing through the stiff wire. The MitraClip XTW was inserted into the catheter with a 90° counterclockwise rotation. After adjusting to a straddle position to move the clip laterally, additional knob rotations were performed to position the clip at A2/P2. Once the clip was placed, only trivial mitral regurgitation (MR) remained. No complications occurred, and the patient improved, allowing discharge. Transthoracic echocardiography at 1-year post-procedure demonstrated sustained MR reduction.

**Discussion:**

We have described the successful completion of M-TEER using the RIJ approach in a patient with severe FMR. Technical considerations in M-TEER require special attention because of limited reports on the M-TEER procedure via the RIJ.

**Learning points**

- The right internal jugular approach offers a feasible alternative for performing transcatheter edge-to-edge mitral valve repair with MitraClip when femoral access is not possible.
- To provide a backup for the steerable guiding catheter (SGC), a stiff wire was used to facilitate advancement into the left ventricle. To position the clip delivery sheath laterally, after adjusting to a straddle position, further knob rotations were employed because simple pushing was insufficient to move the SGC laterally.

## Introduction

In patients with symptomatic functional or degenerative mitral regurgitation (MR) at prohibitive surgical risk, transcatheter edge-to-edge mitral valve repair (M-TEER) with the MitraClip (Abbott Medical, CA, USA) or PASCAL (Edwards Lifesciences, CA, USA) system offers a viable option.^1,2^ The absence of transfemoral access is a contraindication. However, cases of inadequate transfemoral access are emerging. MitraClip procedures are performed in patients with occluded inferior vena cava (IVC) filters^3,4^ and severely tortuous iliac veins using the right internal jugular (RIJ) approach.^5^ Given limited reports, additional adjustments may be necessary, depending on the patient’s anatomy. Herein, we report successful M-TEER via the RIJ approach in a patient with functional MR, using previously undescribed techniques.

## Summary figure

Step-by-step procedure of M-TEER using the MitraClip system via the RIJ approach. Transseptal puncture performed through the RIJ using the SupraCross™ steerable sheath and its radiofrequency wire (Boston Scientific, Natick, MA, USA), targeting a mid-posterior septal position 32 mm above the mitral annulus (*A*). The SupraCross™ and steerable guiding catheter (SGC) collapsed towards the right ventricle (RV) and could not be advanced into the left atrium (LA; *B* and *C*). To provide backup support, a 5-French pigtail catheter was manoeuvred from the A2/P2 segment into the left ventricle (LV) to avoid entanglement with the mitral subvalvular apparatus (*D* and *E*), and a Confida wire (Medtronic, Minneapolis, MN, USA) was positioned at the bottom of the LV (*F* and *G*). By manoeuvring the knobs to achieve the straddle position (*H*), the MitraClip XTW was successfully deployed at the A2/P2 scallop (*I* and *J*).

**Figure:**
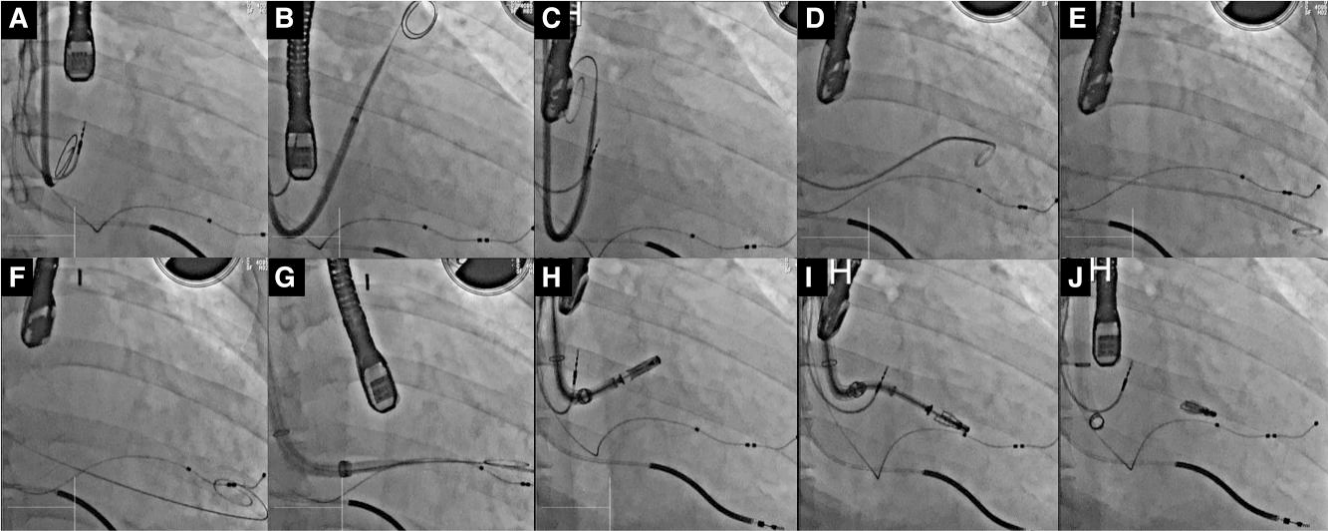


## Case presentation

**Figure 1:**
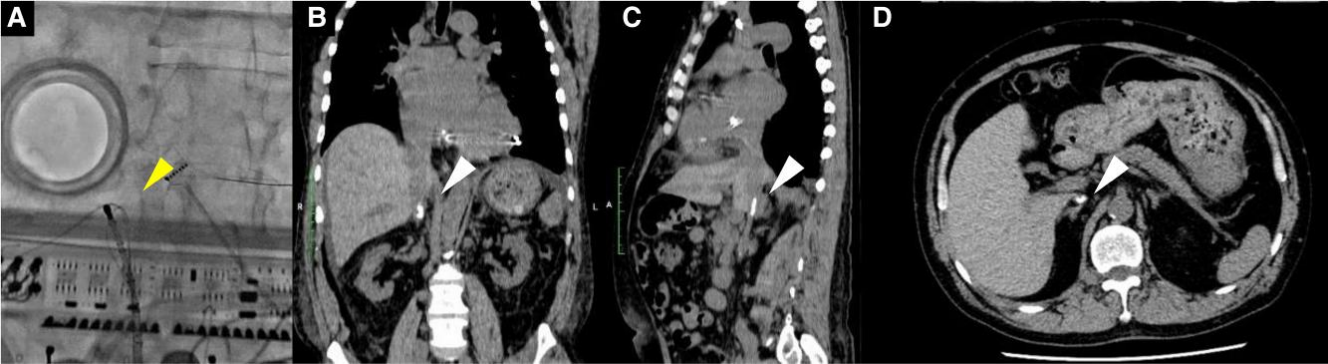
Fluoroscopy (*A*) and computed tomography (*B–D*) revealed that both the radiofrequency ablation catheter and the 0.035 in spring coil wire could not advance from the femoral vein to the right atrium due to calcified venous thrombosis blocking the inferior vena cava at the renal vein level. The white arrowhead indicates the radiofrequency ablation catheter and the 0.035 in spring coil wire, while the white arrowhead shows calcified venous thrombosis.

**Figure 2:**
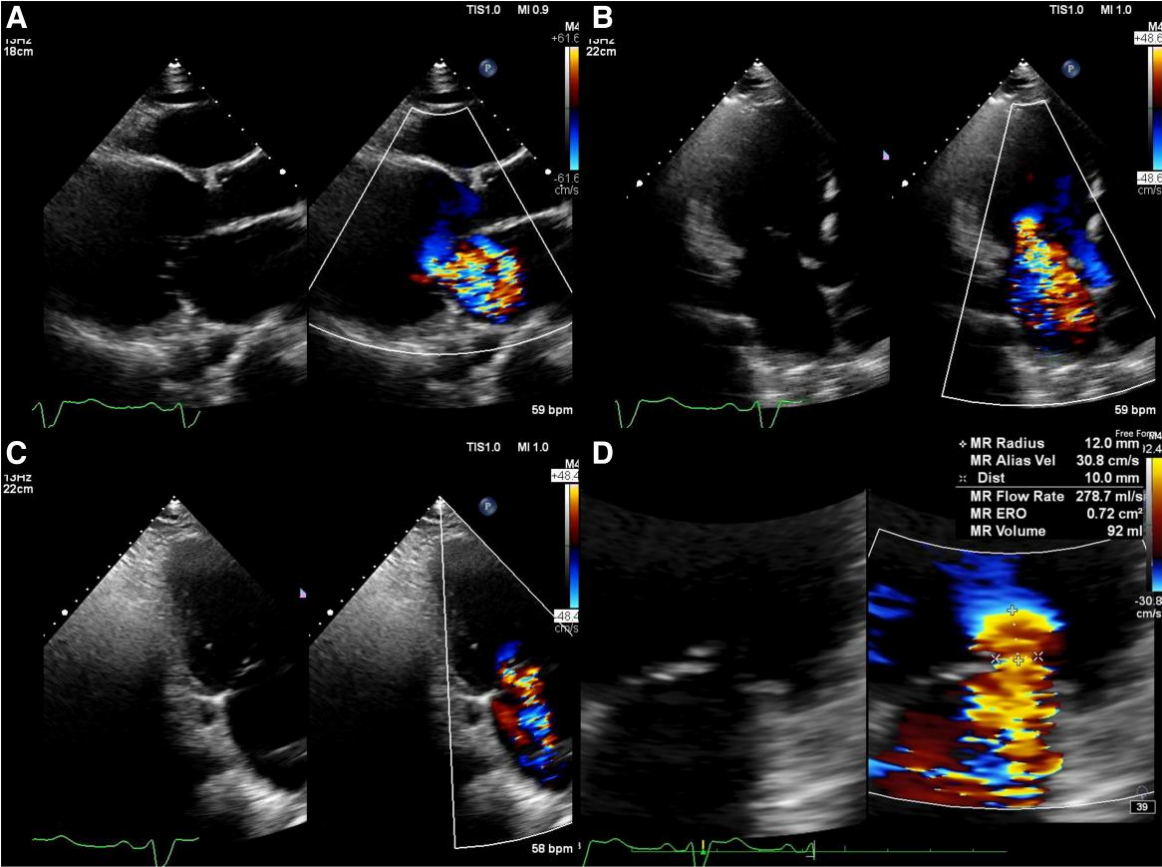
Preprocedural transthoracic echocardiography images show severe mitral regurgitation from A2/P2 (*A–C*). The quantification of mitral regurgitation by the Proximal Isovelocity Surface Area method showed an effective regurgitant orifice area of 72 mm^2^ and a regurgitant volume of 92 mL (*D*).

The patient was a 57-year-old man with worsening exertional dyspnoea [New York Heart Association (NYHA) class IV], a history of dilated cardiomyopathy, a cardiac resynchronization therapy defibrillator implanted for ventricular tachycardia, and pulmonary embolism secondary to deep vein thrombosis. A year ago, a femoral vein radiofrequency ablation attempt for ventricular tachycardia was unsuccessful as the catheter could not be advanced into the right atrium (*Figure 1A*). Computed tomography revealed IVC occlusion from calcified venous thrombosis (*Figure 1B–D*). Transthoracic echocardiography (TTE) showed a reduced ejection fraction (35%) and severe functional MR at A2/P2 (Carpentier type I and effective regurgitant orifice area, 72 mm^2^; *Figure 2*). The TTE findings were as follows: LV diameter in diastole/in systole, 86/73 mm; LV end-diastolic volume index, 153 mm/m^2^; LA volume index, 56 mL/m^2^; and systolic pulmonary artery pressure, 40 mmHg. The tricuspid annular plane systolic excursion and fractional area change were 15.7 mm and 22%, respectively, indicating impaired RV function. Transoesophageal echocardiography (TEE) demonstrated the following: septal puncture height, 32 mm; mitral valve area, 8.2 cm^2^; posterior leaflet length at P2, 16 mm; and no coaptation length. The patient, with a history of dilated cardiomyopathy, presented with severe NYHA class IV heart failure that was unresponsive to optimal medical therapy (enalapril 5 mg/day, bisoprolol 5 mg/day, dapagliflozin 10 mg/day, and spironolactone 50 mg/day), intravenous treatment, and mechanical circulatory support, including intra-aortic balloon pumping. Despite these interventions, the heart failure remained refractory, accompanied by cardiogenic shock (systolic blood pressure of 86 mmHg and heart rate of 110 b.p.m.). After the heart team discussion, the patient was deemed high risk for surgical mitral valve replacement, as reflected by high scores in the Society of Thoracic Surgeons Predicted Risk of Mortality (17.1%), EuroSCORE II (18.6%), and COAPT risk assessment (11). The decision was made to pursue transjugular M-TEER using the MitraClip system as an alternative, less invasive alternative.

**Figure 3:**
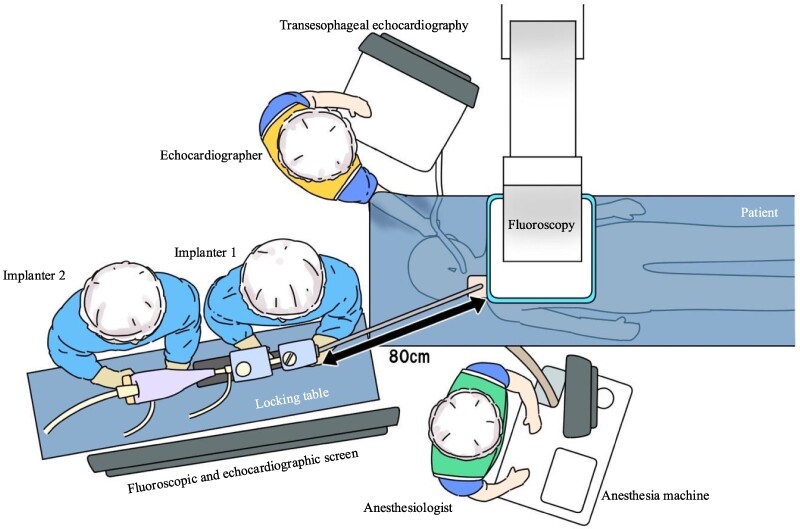
Schematic illustration of room setup for transcatheter edge-to-edge mitral valve repair via the right internal jugular vein. This approach positions the implanters at the patient’s head on their right side, with an additional table 80 cm from the chest for the MitraClip stabilizer and display screens for fluoroscopy and transoesophageal echocardiography in front of the physicians. A locking table is essential for the stabilizer. The echocardiographer is positioned on the patient’s left, while the anaesthetist is at the foot of the bed.

**Figure 4:**
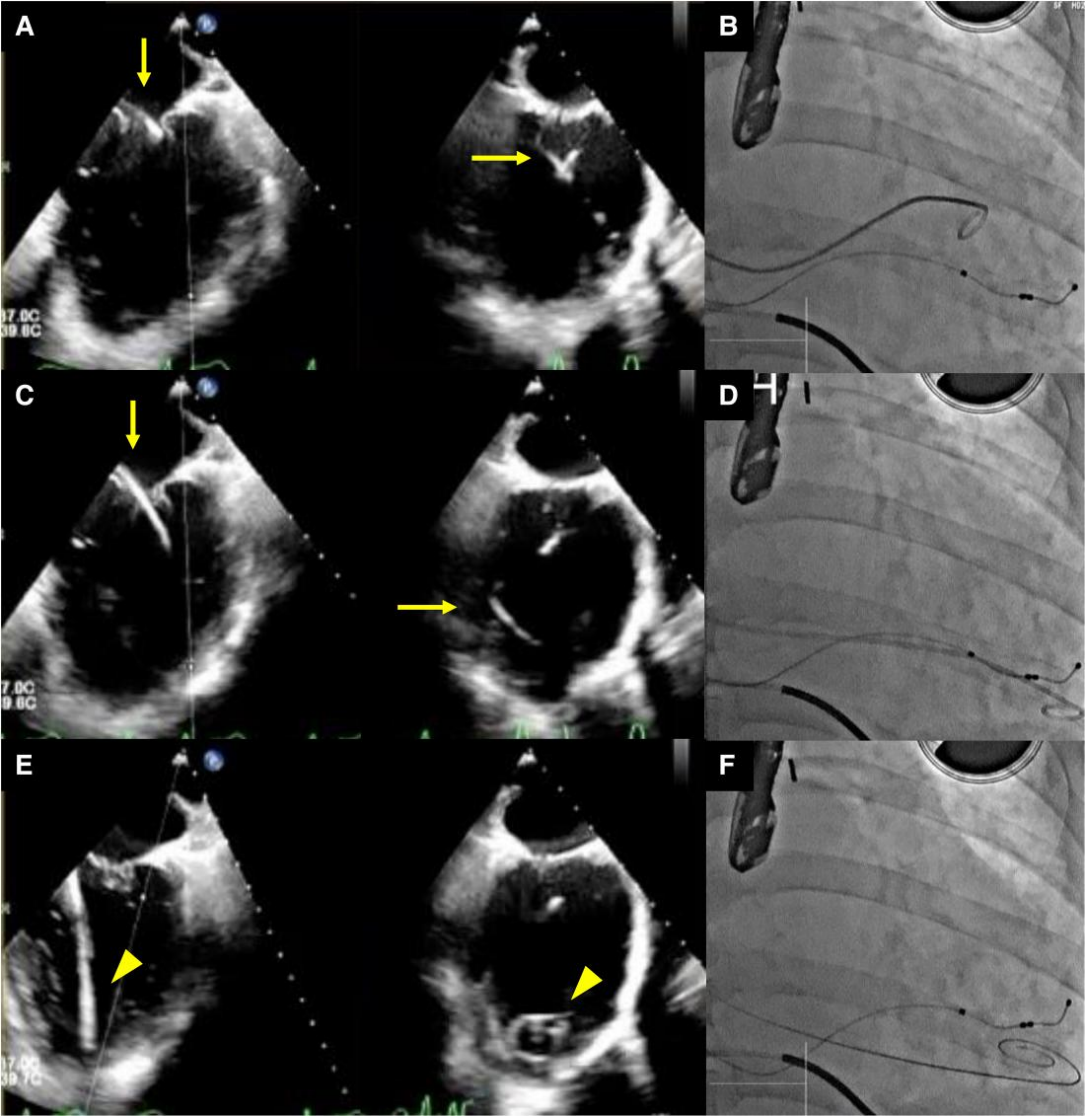
Transoesophageal echocardiography images (*A*, *C*, and *E*) and concurrent fluoroscopy images (*B*, *D*, and *F*) are shown. The left-side images display the bicommissural view, while those on the right side illustrate the left ventricular outflow tract view in transoesophageal echocardiography. Guided by transoesophageal echocardiography, a 5-French pigtail catheter was advanced through the mitral valve at the A2/P2 scallop into the left ventricle using a coil wire (*A*, bicommissural view, and *B*), ensuring it did not entangle with the chordae tendineae as it was directed towards the apex (*C* and *D*). The catheter was subsequently replaced with a Confida wire, and the tip was confirmed at the apex, enabling the safe insertion of the guiding catheter (*E* and *F*). In the transoesophageal echocardiography images, the arrow identifies the pigtail catheter, while the arrowhead points to the Confida wire.

*Figure 3* details the room setup and positioning for the RIJ approach. The RIJ was cannulated under ultrasound guidance, and one Perclose (Abbott Medical, CA, USA) was deployed in the preclose technique. Transseptal puncture was performed via the RIJ using the SupraCross™ system in a mid-posterior septal position, 32 mm above the mitral valve annulus. To facilitate septal crossing, the SGC was advanced with a 180° rotation of the +knob. Since simple pushing failed, we performed balloon septostomy using MUSTANG 8.0 × 40 mm (Boston Scientific, MA, USA), but the SGC still did not cross the septum. To avoid wire entanglement with the mitral subvalvular apparatus, a 5-French pigtail catheter over a 0.035 in spring coil wire (Medikit, Tokyo, Japan) was manoeuvred from A2/P2 into the LV under TEE guidance (*Figure 4*). A Confida wire was placed in the LV to support SGC crossing the septum. The clip delivery sheath (CDS) was mis-keyed at a 90° counterclockwise rotation. In the under-straddle position, knob manipulations were ineffective for lateral advancement and required additional use of the A-knob. The +knob, A knob, and M knob were turned 180° for height, 180° for lateral movement, and 270° to advance the CDS to A2/P2, respectively.

**Figure 5:**
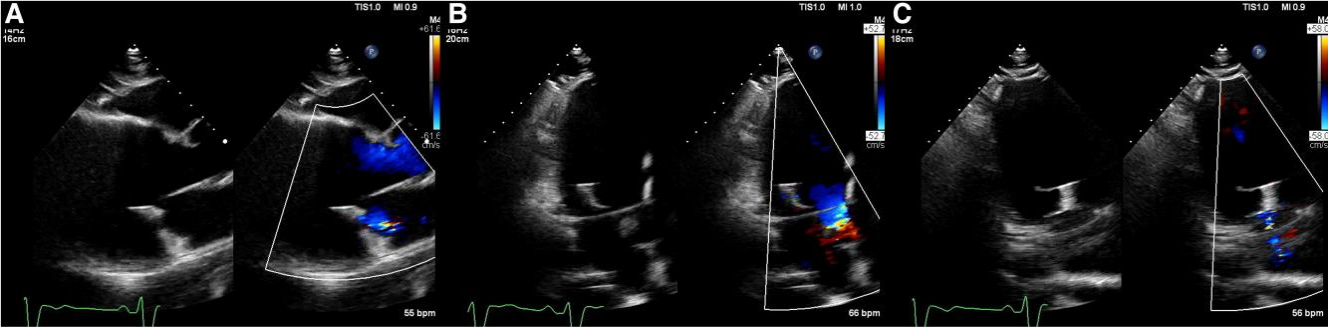
Postprocedural transthoracic echocardiography images showed mild residual mitral regurgitation (*A–C*).

Once adequately positioned, the remaining steps of M-TEER with the MitraClip via the RIJ were similar to those of the femoral approach. After deploying a MitraClip XTW at A2/P2, trace MR with a mean gradient of 2 mmHg was observed. The procedural time was 182 min without complications. The patient improved and was discharged. Postprocedural TTE showed reduced MR (*Figure 5*). At 1-month follow-up, the NYHA class was I, TTE showed mild MR, and NT-proBNP decreased from 10 540 to 1407 pg/mL. One year later, the NYHA class was unchanged, TTE showed trivial MR, and NT-proBNP was 612 pg/mL, with no heart failure hospitalizations. We are considering heart transplant or using a long-term left ventricular assist device later.

## Discussion

Herein, two key points are highlighted: effective use of the Confida wire to facilitate SGC advancement and the importance of adopting a straddle position for optimal clip placement.

Unlike the femoral vein approach, during transseptal puncture via the RIJ route, a clockwise torque of the transseptal sheath causes anterior movement, while a counterclockwise torque causes posterior movement. The transseptal sheath should be positioned in the IVC and retracted with the indicator at about 8 o’clock. The use of a steerable sheath may aid in accurate interatrial septum positioning when employing the transjugular route.^5,6^ The +knob gains height and moves laterally, while the −knob loses height and moves medially, resulting in opposite movements.^5,7^

We summarized the M-TEER procedure using the MitraClip system via the RIJ in Supplementary material online, *Table S1*, highlighting differences from earlier reports. When the CDS was in the under-straddle position, knob manipulation was ineffective for lateral positioning. Previously, once the clip entered the LA, it was advanced and kept in the ‘under-straddled’ position,^3,4,5^ not allowing lateral clip positioning. We initially adjusted the CDS to the straddle position to improve knob manipulation effectiveness. A transseptal height of ≥45 mm is unnecessary.^5^ However, adequate space and height within the LA are required to achieve the straddle position.^3^ In this case, a small LA limited the transseptal height to 32 mm, preventing CDS adjustment to the straddle position. Even with the RIJ approach, sufficient LA volume and transseptal height are required to achieve the straddle position. Therefore, the +knob was increased for height. Subsequently, lateral CDS positioning was achieved by knob rotation. Total rotation involved a 180° turn of the +knob, followed by a 270° turn of the M knob, and an additional 180° turn of the A knob. Therefore, unlike the femoral approach, lateral lesions (e.g. A1/P1) may pose challenges due to the inability to reposition laterally using the stabilizer alone.

Finally, the RIJ approach is an alternative to M-TEER with MitraClip when the femoral vein is inaccessible. This technique may be beneficial for patients with a small LA and insufficient septal puncture height.

## Supplementary Material

ytae668_Supplementary_Data

## Data Availability

The data underlying this article will be shared on reasonable request to the corresponding author.

## Lead author biography

Dr Yusuke Enta started his career in Sendai Kousei Hospital, Miyagi, Japan. He is an interventional cardiologist and a specialist in cardiac intervention for structural heart disease including transcatheter edge-to-edge mitral valve repair (M-TEER).
