# Supplementary material for: Transjugular transcatheter edge-to-edge mitral valve repair in a patient with functional mitral regurgitation: a case report
Source: Eur Heart J Case Rep. 2024 Dec 18;9(1):ytae668. doi: 10.1093/ehjcr/ytae668 (PMC11694701; doi:10.1093/ehjcr/ytae668)
Supplement: ytae668_Supplementary_Data [file ytae668_supplementary_data.pdf]

| **Case, year** | **Type of MR and target position** | **Transseptal equipment** | **Transseptal location and height above mitral valve** | **Number of clip** | **CDS position** | **Techniques** | **Procedural Success** | **Complications** |
|---|---|---|---|---|---|---|---|---|
| This case, 2024 | VFMR at A2P2 | SupraCross steerable sheath with radiofrequency wire | Mid-posterior, 32mm | 1 | Straddle position | The SGC was introduced with a 180-degree clockwise rotation of the + knob for septal crossing. The CDS was inserted into the SGC with a 90-degree clockwise rotation. To advance the SGC into the left atrium, the Confida wire was positioned at the bottom of the left ventricle to provide backup. The total rotation involved a 180-degree turn of the + knob, followed by a 270-degree turn of the M knob, and then an additional 180-degree turn of the A knob for navigating the CDS laterally. | Yes | None |
| Fam et al, 2017(3) | DMR at P2 | SupraCross steerable sheath with radiofrequency wire | Mid-posterior, 42mm | 2 | Under-straddle position | The SGC was introduced with a 180-degree clockwise rotation of the + knob for septal crossing. The CDS was inserted into the SGC with a 90-degree clockwise rotation.<br>The MitraClip was navigated into position using the + and P knobs. | Yes | None |
| Chizner et al, 2018(4) | DMR at P2 | TorFlex transseptal guiding sheath and radiofrequency NRG transseptal needle | Superior-posterior, Unknown | 1 | Under-straddle position | The CDS was inserted into the SGC with a 90-degree clockwise rotation. A total rotation involved a 180-degree turn of the + knob, followed by a 150-degree turn of the M knob, and then an additional 180-degree turn of the A knob. | Yes | None |
| Yap et al, 2020(5) | DMR at A3 after surgical mitral valve repair | SupraCross steerable sheath with radiofrequency wire | Superior-mid, Unknown | 1 | Under-straddle position | The CDS was inserted into the SGC with a 90-degree clockwise rotation. | Yes | None |

Detailed techniques from previous reports and the current case regarding the use of the MitraClip via the right internal jugular approach are summarized. The differences between the current and previous cases are highlighted with underline. M-TEER, Transcatheter Edge-to-Edge Mitral Valve Repair, RIJ, right internal jugular; MR, mitral regurgitation; CDS, clip delivery sheath; VFMR, ventricular functional mitral regurgitation; DMR, degenerative mitral regurgitation; SGC, steerable guiding catheter
